# Supplementary material for: Structural insights into the role of GTPBP10 in the RNA maturation of the mitoribosome
Source: Nat Commun. 2023 Dec 2;14:7991. doi: 10.1038/s41467-023-43599-z (PMC10693566; doi:10.1038/s41467-023-43599-z)
Supplement: Supplementary file 1 — Supplementary Information [file 41467_2023_43599_MOESM1_ESM.pdf]

Supplementary information

Structural insights into the role of GTPBP10 in the RNA maturation of the mitoribosome

Thu Giang Nguyen[1], Christina Ritter[1], and Eva Kummer[1]*

[1]Novo Nordisk Foundation Center for Protein Research, University of Copenhagen, Blegdamsvej 3B, 2200 Copenhagen, Denmark

*corresponding author: eva.kummer@cpr.ku.dk

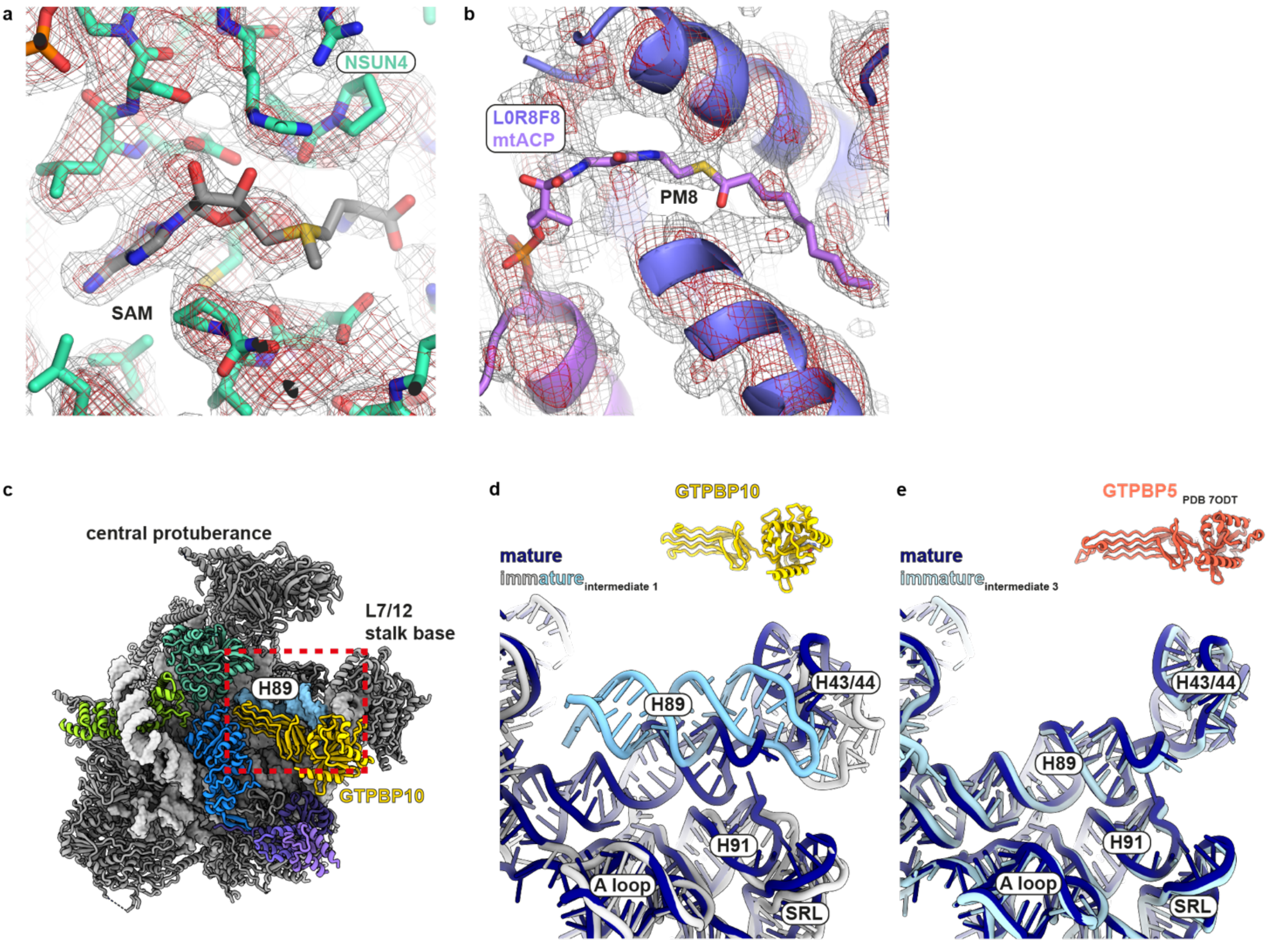

Supplementary Fig. 1 H89 maturation by GTPBP10 and GTPBP7

a) Structural models and experimental EM density is shown for NSUNS4 (cyan) and its co-factor S-adenosyl-methionine (SAM, grey). The sharpened cryo-EM density is shown at two thresholds: 1.5 σ (grey), 2.5 σ (red). **b)** Structural model and experimental EM density is displayed for mtACP and its acyl ligand PM8 (light violet). The sharpened EM density is depicted at two levels: 2 σ (grey) and 3 σ (red). **c)** An overview of the structural model of intermediate 1 is shown with rRNA helix H89 highlighted in sky blue. GTPBP10 (yellow) extensively contacts the helix to stabilize it in a position on top of its crevice on the large mitoribosomal subunit. **d)** Ribosomal RNA from the immature intermediate 1 (light grey) and the mature mitoribosome (PDB 7NSH[1], dark blue) have been superimposed to illustrate that many ribosomal RNA elements have already adopted their final location in intermediate 1 except helix H89 (sky blue). **e)** Superposition of the same ribosomal RNA elements as in panel a) for the maturation intermediate containing the second mitochondrial ObgE homolog GTPBP5 (PDB 7ODT[2], light blue). In this case, H89 is already fully accommodated. SRL = sarcin-ricin loop

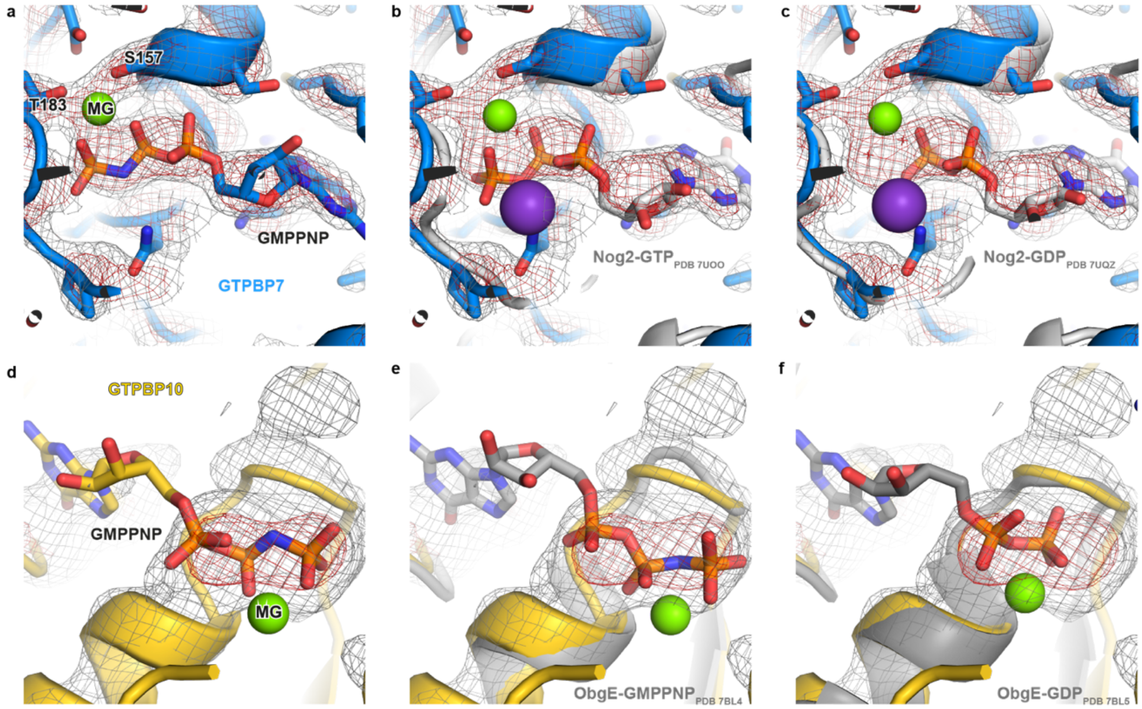

Supplementary Fig. 2 Guanosine nucleotides in the active sites of GTPBP10 and GTPBP7

a) Structural models and experimental EM density is shown for the GTPBP7 active site and the bound nucleotide. Residues that are involved in coordination of a magnesium ion (MG, light green sphere) are highlighted. The sharpened cryo-EM density was 5 times supersampled and is shown as isomesh at two thresholds: 3.0 σ (red) and 2.3 σ (grey). **b and c)** Same as a but the structure of the cytosolic biogenesis factor Nog2 (grey) has been superimposed in PyMOL. GTP (PDB 7UOO)[3] and GDP (PDB 7UQZ)[3] molecules as well as magnesium (light green) and potassium ions (violet) modelled in the Nog2 active site are shown in an isomesh of the experimental density for intermediate 1. Thresholds of the density are the same as for panel a). **d)** Structural model and EM density for the GTPBP10 active site and the bound nucleotide. The sharpened EM density has been supersampled 5 times and is displayed as isomesh at two thresholds: 4.5 σ (red) and 3.2 σ (grey). **e)** Same as panel d) and the GTPase domain of ObgE of PDB 7BL5[4] (grey) including its GDP molecule is superimposed. **f)** Same as panel d) and the GTPase domain of ObgE of PDB 7BL4[4] (grey) including its GMPPNP molecule is superimposed.

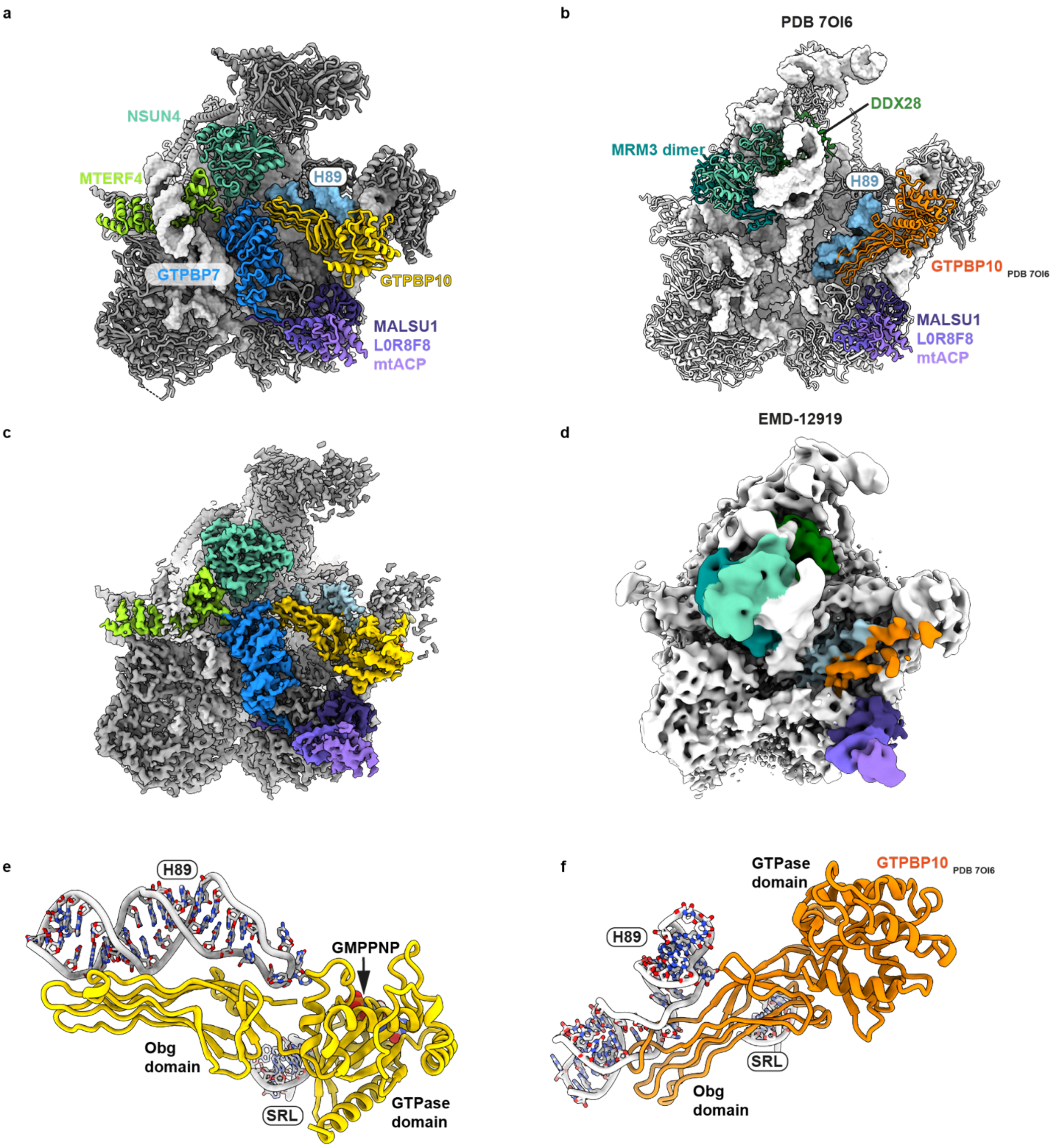

Supplementary Fig. 3 Comparison of intermediate 1 with a previously published intermediate
a and b) Overview of GTPBP10-containing intermediate 1 and the maturation intermediate previously published with the putative GTPBP10 (PDB 7OI6)[5]. The previous publication assigns GTPBP10 (orange) to adopt a distinct conformation on the large ribosomal subunit that is incompatible with GTP hydrolysis and occurs in the absence of maturation factor GTPBP7 at a distinct step in the maturation process. Moreover, no contact to MALSU1 and the GTPBP10 GTPase domain can be seen. **c and d)** Experimental EM maps for the complexes displayed in panels a) and b) color-coded according to the corresponding PDBs. **e and f)** Detailed view of panels a) and b) demonstrating a different interaction of the GTPase domain with the sarcin-ricin loop (SRL) and clashes of GTPBP10 with RNA helix H89 in PDB 7OI6.

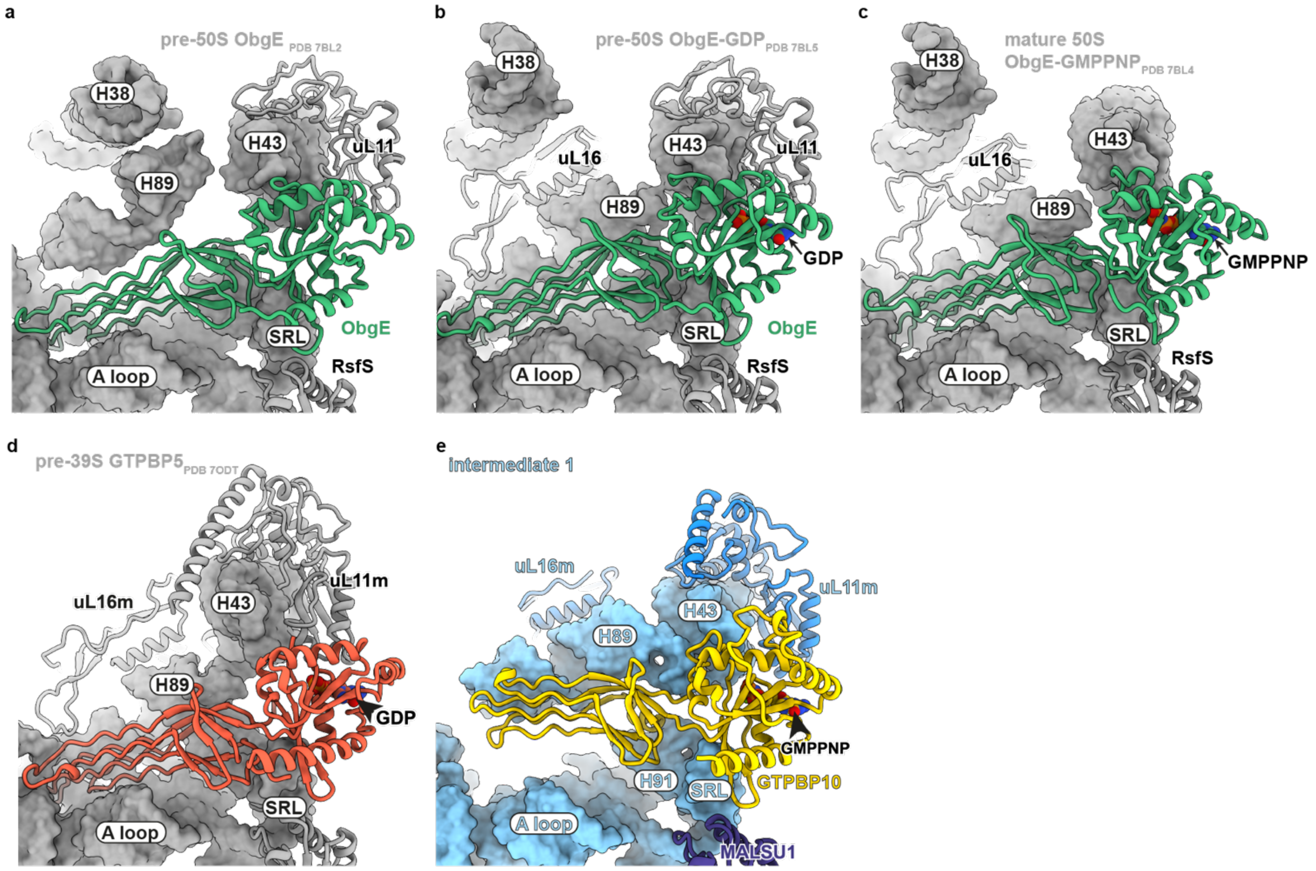

Supplementary Fig. 4 Ribosomal RNA elements in bacterial and mitochondrial ribosome biogenesis
a-c) Depiction of the structural models displaying the steps of H89 maturation by the bacterial GTPase ObgE (green) from *E. coli*.[4] Panel a) and b) show ribosome biogenesis intermediates isolated from *E. coli*. In panel a), H89 is still far from its final position and blocks together with H38 the uL16 binding site. In panel b), H89 has been accommodated allowing for incorporation of uL16 and GTP hydrolysis in ObgE. In panel c), the mature, bacterial 50S has been incubated with ObgE in presence of the non-hydrolysable nucleotide analog GMPPNP. Ribosomal stalk base (with H43) and the GTPase domain of ObgE are in a different, presumably pre-hydrolysis conformation than in panel b). **d and e)** Overview of the position of GTPBP5 and GTPBP10 and the mtLSU interface. Notable is the difference between the location of H89 and the GTPase activating center (GAC) between both intermediates. Important ribosomal RNA elements and mitoribosomal protein uL16m have been labelled. SRL = sarcin-ricin loop

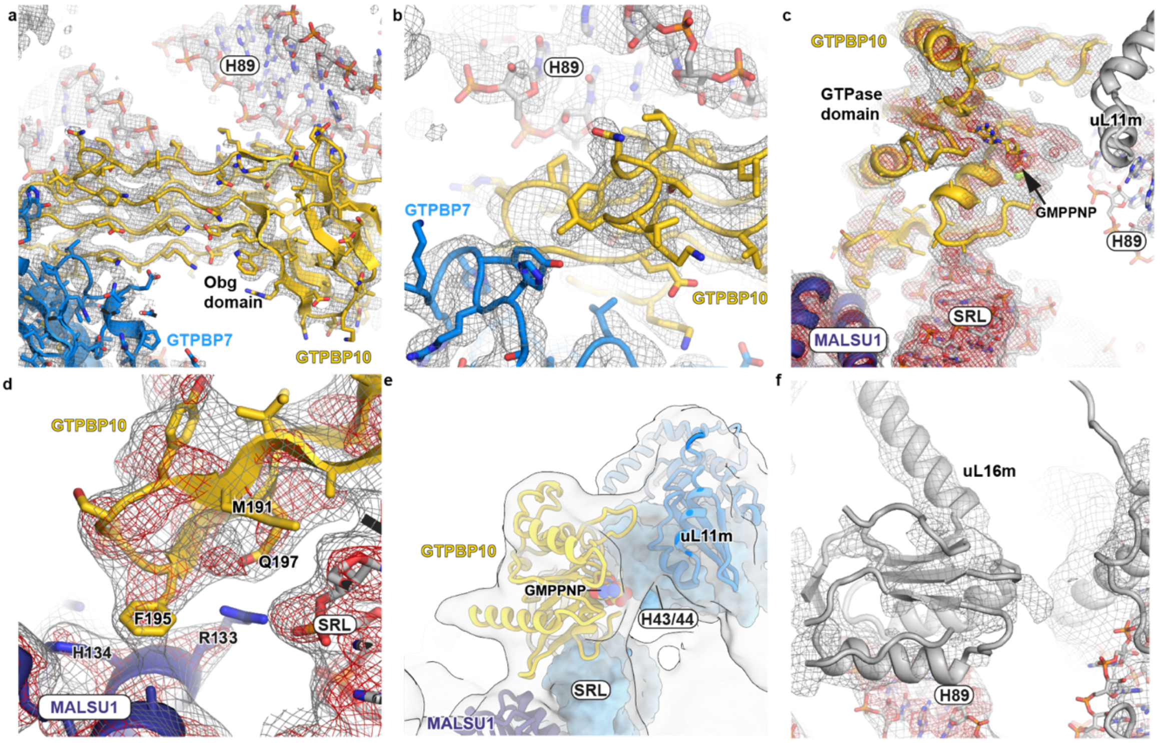

Supplementary Fig. 5 Experimental EM densities for various regions of intermediate 1

a and b) Different magnifications of the same view of the interaction site of GTPBp10 with H89 and GTPBP10 showing how the Obg domain of GTPBP10 clamps between the RNA and GTPBP7. The unsharpened, experimental EM density has been supersampled 5 times and is shown as isomesh at 2.0 σ. **c)** View of the GTPBP10 GTPase domain bound to the SRL using the same settings for the EM density as in panels a) and b). **d)** Close-up of the interaction between the GTPBP10 GTPase domain and biogenesis factors MALSU1. EM density is shown with the same settings as in panels a) and b). **e)** The GTPBP10 GTPase domain is shown in the context of the ribosomal stalk base. The experimental EM density has been gaussian-filtered at 2 σ and is shown as semi-transparent surface at threshold 0.125. f) uL16m is depicted with the corresponding EM density gaussian filtered at sd=1 and displayed at a threshold of 4.5 σ. SRL = sarcin-ricin loop

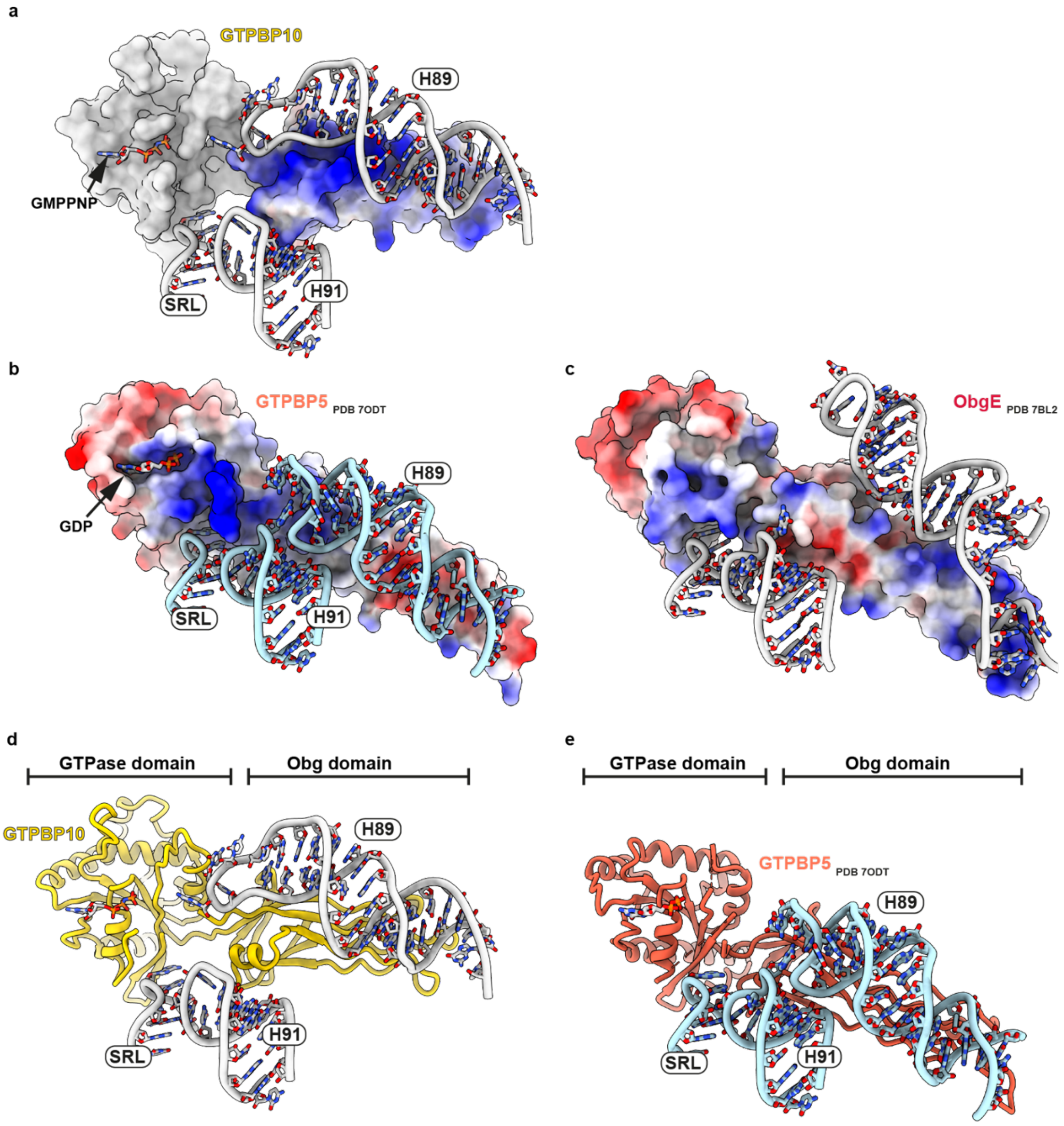

Supplementary Fig. 6 Comparison of GTPBP10, GTPBP5 and bacterial ObgE

a-c) Surface representations of the Obg proteins coloured according to coulombic electrostatic potential using default settings (blue = positive (10), red = negative (-10)) in ChimeraX. Structural models for GTPBP5 and surrounding RNA elements were derived from PDB 7ODT[2], and for ObgE from PDB 7BL2[4]. Surrounding RNA elements including the sarcin-ricin loop (SRL), RNA helix H89, and RNA helix H91 are shown as cartoons. We excluded the GTPase domain of GTPBP10 from the calculation as it has been stripped to poly-alanine in the final model. **d and e)** Structural models for GTPBP10 and GTPBP5 shown in the context of important ribosomal RNA elements. The different position of H89 is apparent as well as the absence of the alpha-helical insertion in the Obg domain of GTPBP5.

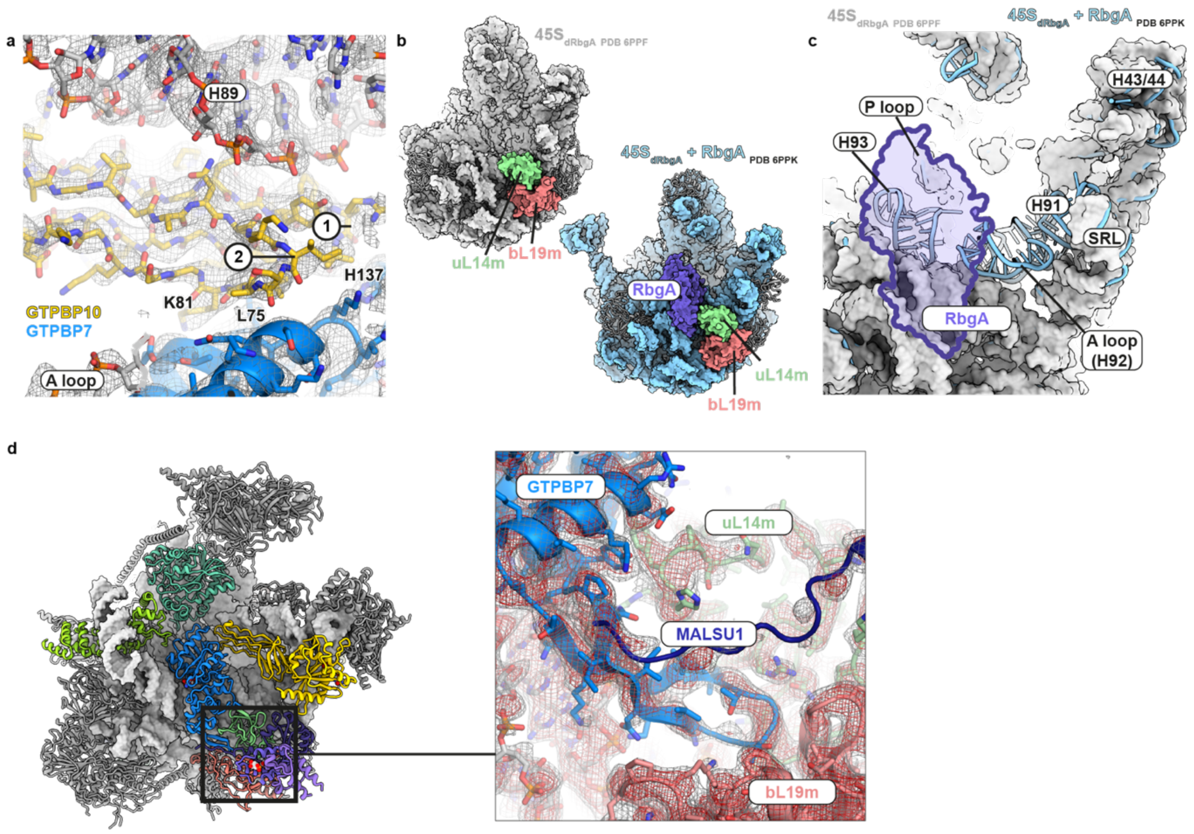

Supplementary Fig. 7 The role of GTPBP7 in H89 maturation

a) A close-up of how GTPBP10 clamps between GTPBP7 and H89 is shown. Loops 1 and 2 of the GTPBP10 Obg domain are labelled. The EM density of the sharpened, 5 times supersampled EM map is displayed at 2.3 σ. **b)** Overview of structural models for a 45S bacterial LSU intermediate isolated from RbgA-depleted cells (grey, PDB 6PPF) and supplemented with recombinant RbgA (light blue, PDB 6PPK). **c)** Overlay of ribosomal RNAs of the 45S intermediate before (grey surface) and after (light blue cartoon) supplementation with RbgA. The binding site of RbgA is shown as silhouette. **d)** Overview of the interaction of GTPBP7 with ribosomal elements. The region boxed in the overview is detailed on the right. A beta-hairpin of GTPBP7 serves to anchor the protein between ribosomal proteins uL14m and bL19m. In addition, the C-terminal tail of MALSU1 reaches over to contact GTPBP7. The EM density is shown at 2 thresholds (red = 3.5 σ, grey = 2.1 σ). SRL = sarcin-ricin loop

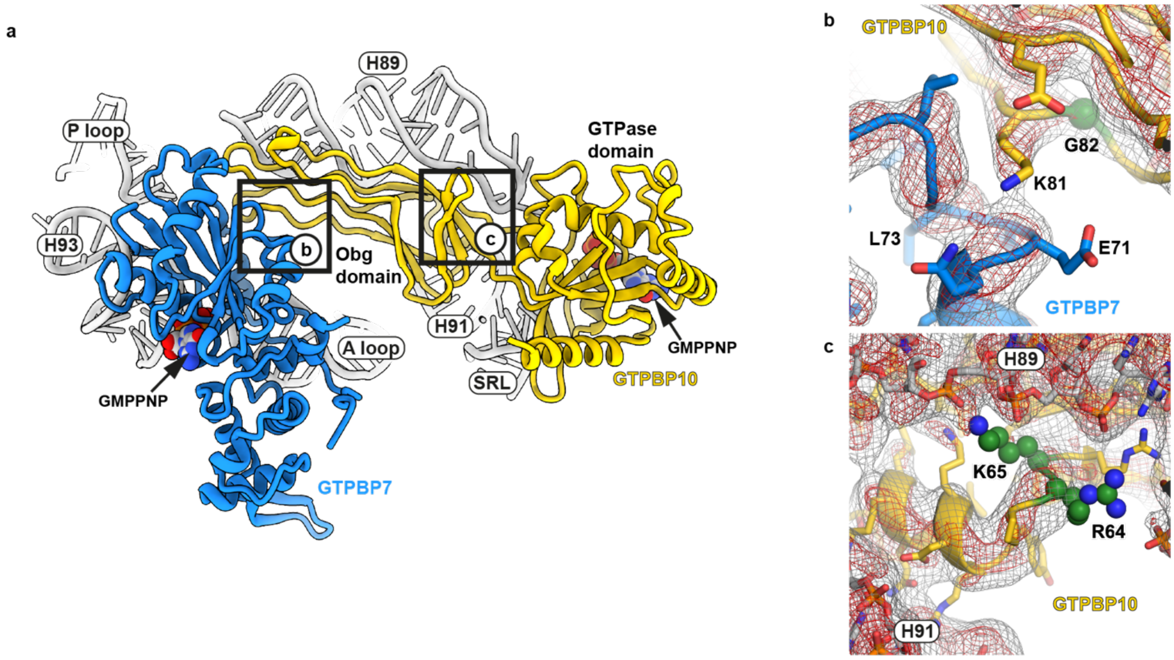

Supplementary Fig. 8 Mutations disturbing GTPBP10 function in mitoribosome biogenesis

a) An overview of GTPBP10 and GTPBP7 in the context of ribosomal RNA is shown and the regions, in which the mutations are located are highlighted with boxes corresponding to the panels shown below. **b and c)** Mutations are highlighted in green and as spheres in the structural model of GTPBP10 with the EM density included. The density is shown for the sharpened, supersampled map at 2 thresholds (red = 3.2 σ, grey = 2.3 σ). SRL = sarcin-ricin loop

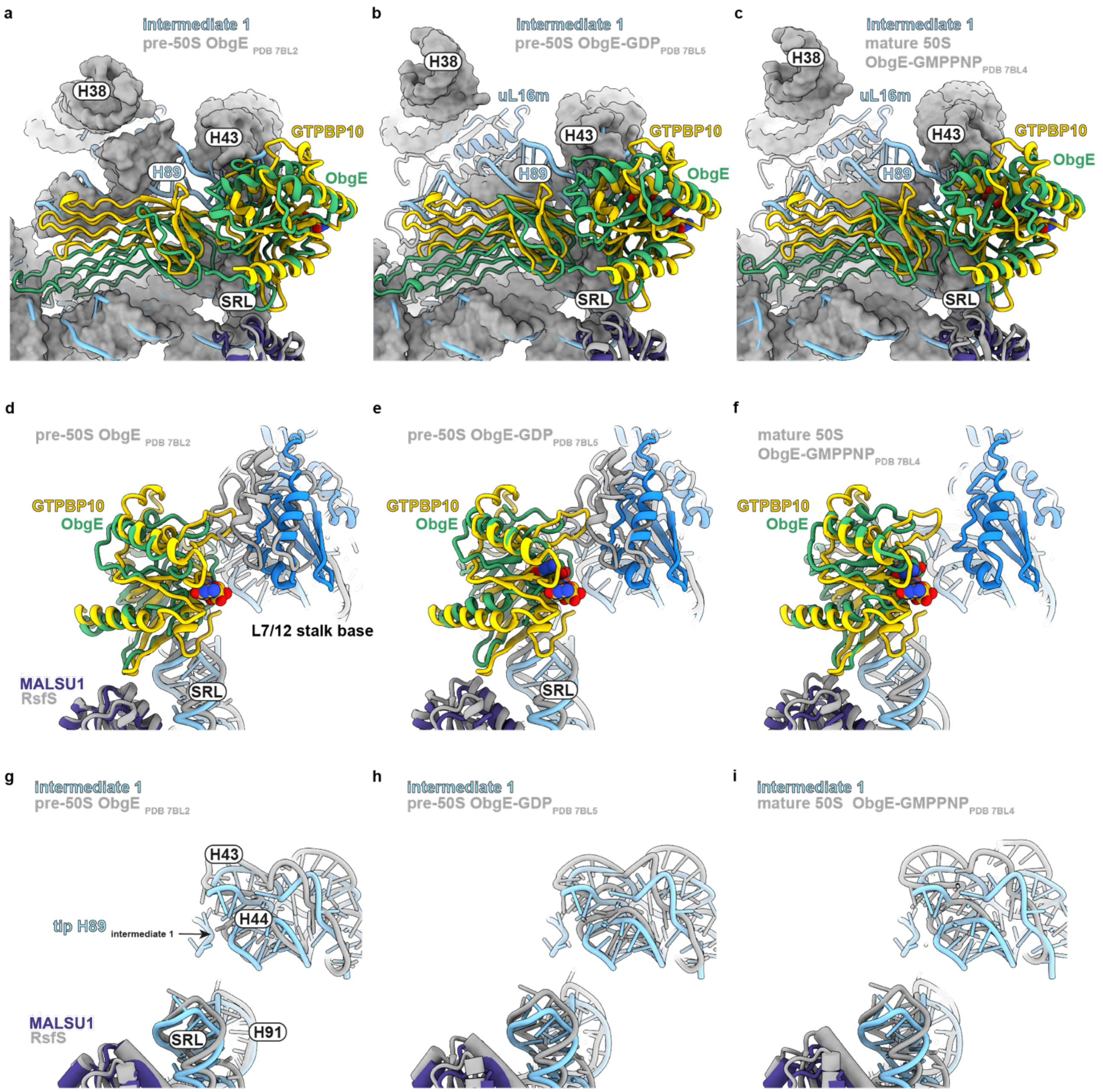

Supplementary Fig. 9 Comparison of intermediate 1 to H89 maturation by ObgE

a-c) Depiction of the structural models displaying the steps of H89 maturation by the bacterial GTPase ObgE (green) from *E. coli*.[4] Panel a) and b) show ribosome biogenesis intermediates isolated from *E. coli*. In panel a), H89 is still far from its final position and blocks together with H38 the uL16 binding site. In panel b), H89 has been accommodated allowing for incorporation of uL16 and GTP hydrolysis in ObgE. In panel c), the mature, bacterial 50S has been incubated with ObgE in presence of the non-hydrolysable nucleotide analog GMPPNP. Ribosomal stalk base (with H43) and the GTPase domain of ObgE are in a different, presumably pre-hydrolysis conformation than in panel b). Intermediate 1 has been superimposed via the ribosomal RNA onto the E. coli structures. Bacterial ribosomal RNA is shown as grey surface representation. The RNA of the mitochondrial intermediate and mitochondrial uL16m are shown as light blue cartoon. GTPBP10 is yellow and the mitochondrial biogenesis factor MALSU1 is dark violet. The conformation of H89 in mitochondrial intermediate 1 matches the one in PDB 7BL2 the best. The Obg domain of GTPBP10 adopts a distinct conformation on the ribosomal RNA than the Obg domain of ObgE. For clarity, the ribosomal proteins surrounding the L7/12 stalk base have been omitted. **d-f)** Essentially the same as panels a-c), but shown from a different angle highlighting the association of the GTPase domain to the SRL and the L7/12 stalk base. Here, ribosomal protein uL11 or uL11m for the bacterial or mitochondrial ribosome,

respectively, has been included in the image to give an impression of the distinct location of the stalk base in mitoribosomal intermediate 1. **g-i)** Same as panels d-f) but the ObgE and uL11 or GTPBP10 and uL11m have been omitted from the image to clearly show the localization of the surrounding RNA elements from the bacterial (grey) and mitochondrial (light blue) ribosome.

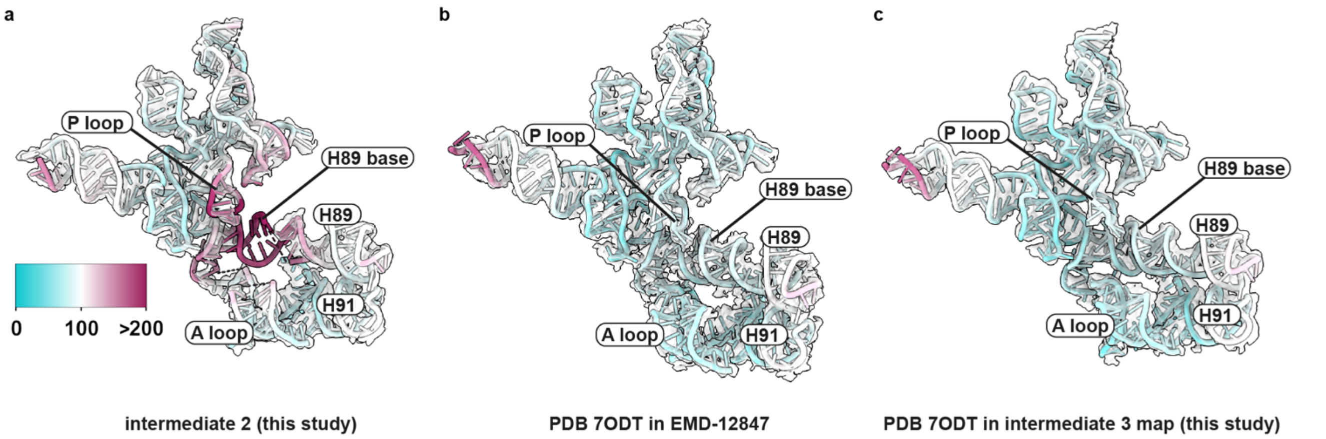

Supplementary Fig. 10 H89 base conformation in intermediates 2 and 3

a-c) The figure shows region 2700-3050 of 16S rRNA from intermediate 2 and PDB 7ODT, which correspond to our intermediate 3 EM map. PDB 7ODT is shown in the experimental EM map from Lenarcic et al.[2] in panel b) and in our intermediate 3 EM density in panel c) indicating that in both cases the experimental density for the base of H89 is clearly defined. The rRNA has been coloured according to B-factors, which we obtained from Phenix real-space refinement (for intermediate 1) or from the deposited PDB 7ODT. B-factors are plotted in ChimeraX-1.5 and the corresponding colour key is shown. Experimental EM density has been carved around the displayed regions with a distance of 3 Å. B factors clearly indicate that the H89 base is still flexible and immature in intermediate 2.

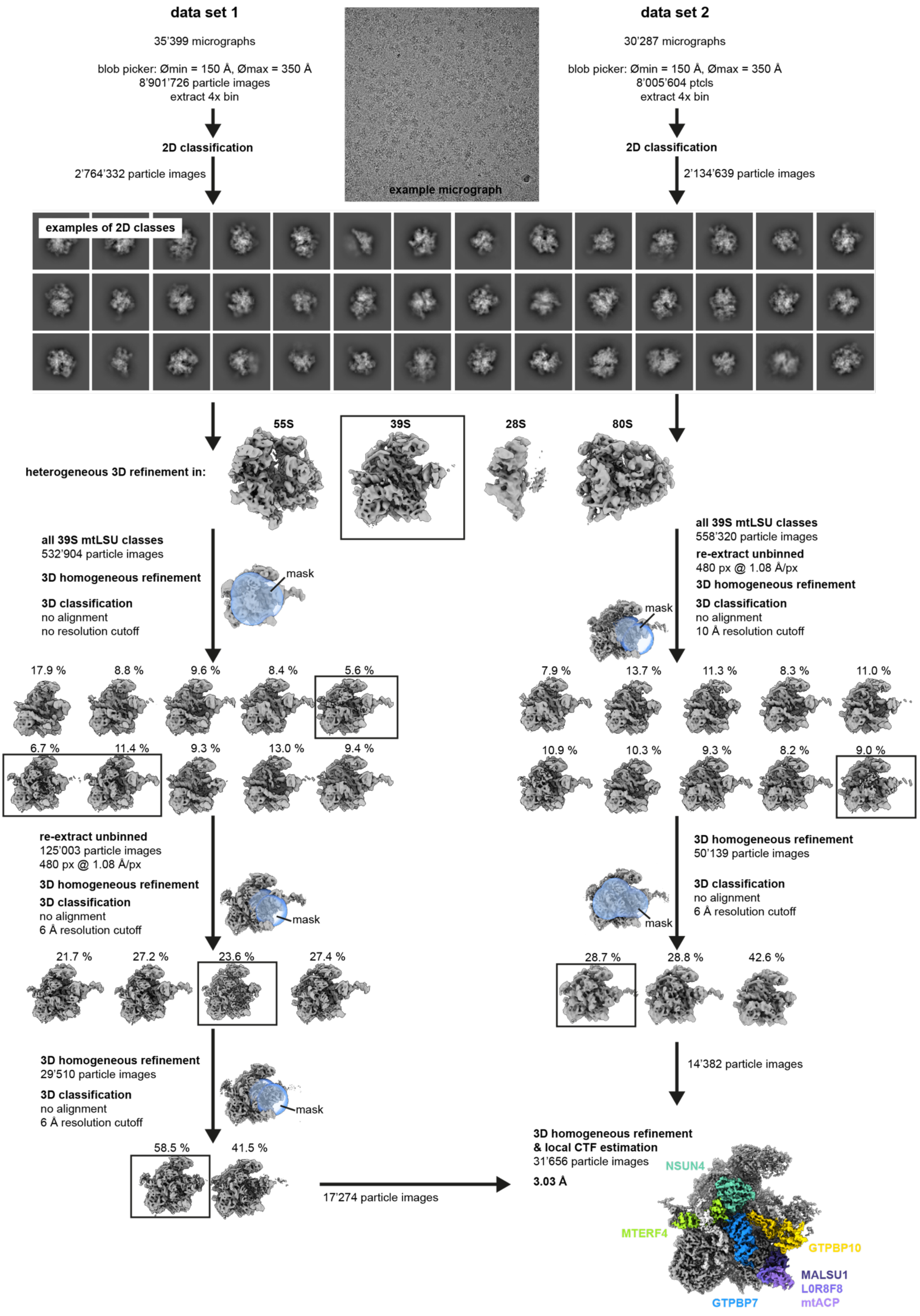
data set 1
35'399 micrographs
blob picker: Ømin = 150 Å, Ømax = 350 Å
8'901'726 particle images
extract 4x bin
2D classification
2'764'332 particle images
example micrograph
data set 2
30'287 micrographs
blob picker: Ømin = 150 Å, Ømax = 350 Å
8'005'604 ptcls
extract 4x bin
2D classification
2'134'639 particle images
examples of 2D classes
heterogeneous 3D refinement in:
55S
39S
28S
80S
all 39S mtLSU classes
532'904 particle images
3D homogeneous refinement
3D classification
no alignment
no resolution cutoff
mask
17.9 %
8.8 %
9.6 %
8.4 %
5.6 %
6.7 %
11.4 %
9.3 %
13.0 %
9.4 %
re-extract unbinned
125'003 particle images
480 px @ 1.08 Å/px
3D homogeneous refinement
3D classification
no alignment
6 Å resolution cutoff
mask
21.7 %
27.2 %
23.6 %
27.4 %
3D homogeneous refinement
29'510 particle images
3D classification
no alignment
6 Å resolution cutoff
mask
58.5 %
41.5 %
17'274 particle images
all 39S mtLSU classes
558'320 particle images
re-extract unbinned
480 px @ 1.08 Å/px
3D homogeneous refinement
3D classification
no alignment
10 Å resolution cutoff
mask
7.9 %
13.7 %
11.3 %
8.3 %
11.0 %
10.9 %
10.3 %
9.3 %
8.2 %
9.0 %
3D homogeneous refinement
50'139 particle images
3D classification
no alignment
6 Å resolution cutoff
mask
28.7 %
28.8 %
42.6 %
14'382 particle images
3D homogeneous refinement
& local CTF estimation
31'656 particle images
3.03 Å
NSUN4
MTERF4
GTPBP10
MALSU1
L0R8F8
mtACP
GTPBP7

Supplementary Fig. 11 Classification scheme for intermediate 1
The classification scheme to derive intermediate 1 containing GTPBP10 and GTPBP7 from datasets 1 and 2 is given. An example micrograph is shown on the top. Mask that were used for local classification are shown in blue and semi-transparent. Classes that were selected for further processing are boxed. Parameters used for local 3D classification ae indicated next to the arrows of the workflow. The final EM reconstruction is shown as unsharpened map and with density corresponding to the biogenesis factors colour-coded.

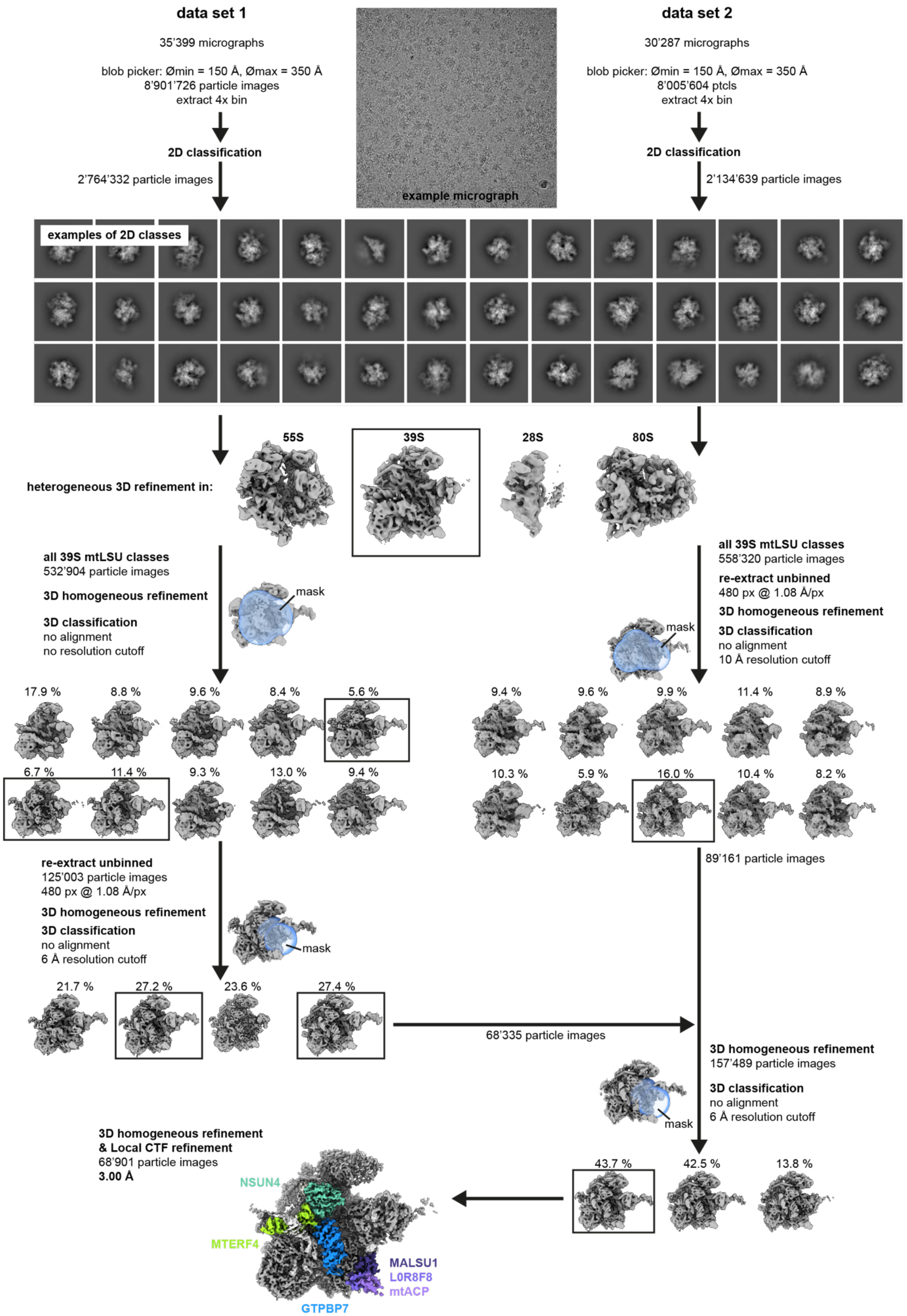

data set 1
35'399 micrographs
blob picker: Ømin = 150 Å, Ømax = 350 Å
8'901'726 particle images
extract 4x bin
2D classification
2'764'332 particle images
example micrograph
data set 2
30'287 micrographs
blob picker: Ømin = 150 Å, Ømax = 350 Å
8'005'604 ptcls
extract 4x bin
2D classification
2'134'639 particle images
examples of 2D classes
heterogeneous 3D refinement in:
55S
39S
28S
80S
all 39S mtLSU classes
532'904 particle images
3D homogeneous refinement
3D classification
no alignment
no resolution cutoff
mask
all 39S mtLSU classes
558'320 particle images
re-extract unbinned
480 px @ 1.08 Å/px
3D homogeneous refinement
3D classification
no alignment
10 Å resolution cutoff
17.9 % 8.8 % 9.6 % 8.4 % 5.6 %
6.7 % 11.4 % 9.3 % 13.0 % 9.4 %
9.4 % 9.6 % 9.9 % 11.4 % 8.9 %
10.3 % 5.9 % 16.0 % 10.4 % 8.2 %
re-extract unbinned
125'003 particle images
480 px @ 1.08 Å/px
3D homogeneous refinement
3D classification
no alignment
6 Å resolution cutoff
89'161 particle images
21.7 % 27.2 % 23.6 % 27.4 %
68'335 particle images
3D homogeneous refinement
157'489 particle images
3D classification
no alignment
6 Å resolution cutoff
3D homogeneous refinement
& Local CTF refinement
68'901 particle images
3.00 Å
NSUN4
MTERF4
GTPBP7
MALSU1
L0R8F8
mtACP
43.7 % 42.5 % 13.8 %

Supplementary Fig. 12 Classification scheme for intermediate 2

The classification scheme to derive intermediate 2 containing GTPBP7 from datasets 1 and 2 is given. An example micrograph is shown on the top. Mask that were used for local classification are shown in blue and semi-transparent. Classes that were selected for further processing are boxed. Parameters used for local 3D classification ae indicated next to the arrows of the workflow. The final EM reconstruction is shown as unsharpened map and with density corresponding to the biogenesis factors colour-coded.

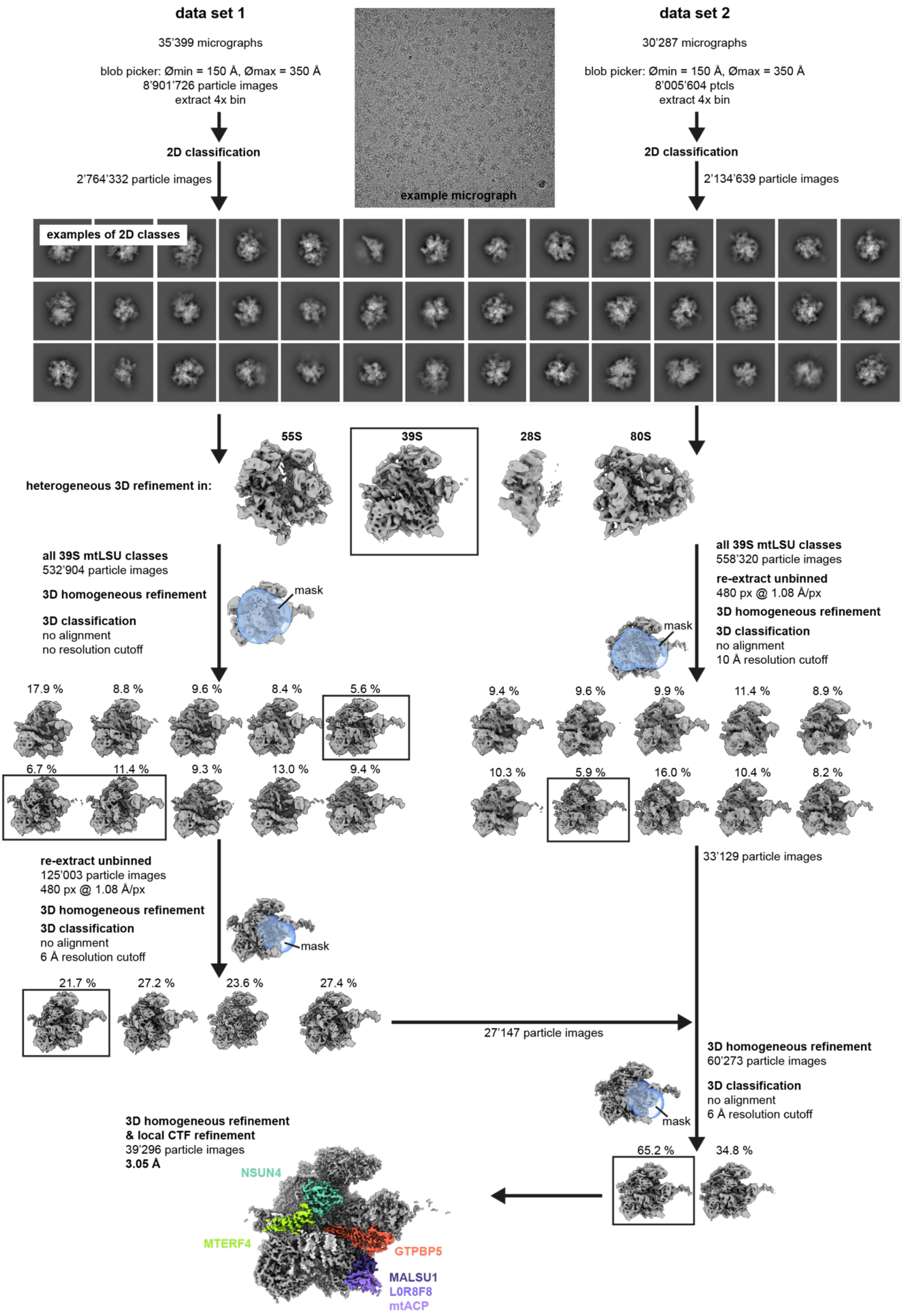
data set 1
35'399 micrographs
blob picker: Ømin = 150 Å, Ømax = 350 Å
8'901'726 particle images
extract 4x bin
2D classification
2'764'332 particle images
example micrograph
data set 2
30'287 micrographs
blob picker: Ømin = 150 Å, Ømax = 350 Å
8'005'604 ptcls
extract 4x bin
2D classification
2'134'639 particle images
examples of 2D classes
heterogeneous 3D refinement in:
55S
39S
28S
80S
all 39S mtLSU classes
532'904 particle images
3D homogeneous refinement
3D classification
no alignment
no resolution cutoff
mask
all 39S mtLSU classes
558'320 particle images
re-extract unbinned
480 px @ 1.08 Å/px
3D homogeneous refinement
3D classification
no alignment
10 Å resolution cutoff
mask
17.9 %
8.8 %
9.6 %
8.4 %
5.6 %
6.7 %
11.4 %
9.3 %
13.0 %
9.4 %
9.4 %
9.6 %
9.9 %
11.4 %
8.9 %
10.3 %
5.9 %
16.0 %
10.4 %
8.2 %
re-extract unbinned
125'003 particle images
480 px @ 1.08 Å/px
3D homogeneous refinement
3D classification
no alignment
6 Å resolution cutoff
mask
33'129 particle images
21.7 %
27.2 %
23.6 %
27.4 %
27'147 particle images
3D homogeneous refinement
60'273 particle images
3D classification
no alignment
6 Å resolution cutoff
mask
3D homogeneous refinement
& local CTF refinement
39'296 particle images
3.05 Å
NSUN4
MTERF4
GTPBP5
MALSU1
L0R8F8
mtACP
65.2 %
34.8 %

Supplementary Fig. 13 Classification scheme for intermediate 3

The classification scheme to derive intermediate 3 containing GTPBP5 from datasets 1 and 2 is given. An example micrograph is shown on the top. Mask that were used for local classification are shown in blue and semi-transparent. Classes that were selected for further processing are boxed. Parameters used for local 3D classification ae indicated next to the arrows of the workflow. The final EM reconstruction is shown as unsharpened map and with density corresponding to the biogenesis factors colour-coded.

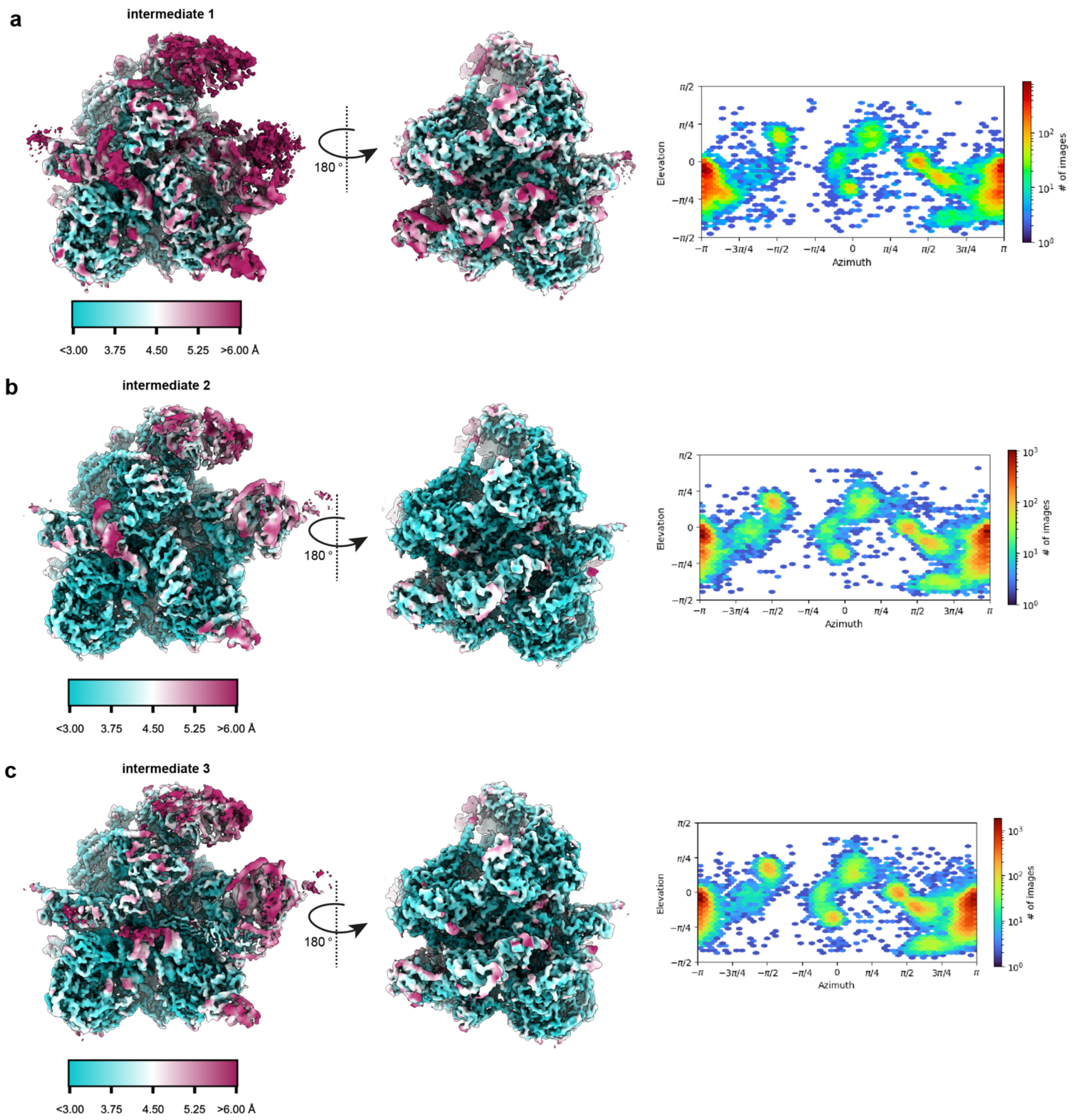

Supplementary Fig. 14 Local resolution and angular distribution

Data for intermediate 1, 2 and 3 are shown in panels **a)**, **b)**, and **c)**, respectively. Local resolution has been estimated in CryoSPARC and is plotted according the the given colour key. A 2D representation of the angular distribution of the particle images in the final reconstruction is given in form of an elevation/Azimuth heatmap (provided in radians, CryoSPARC). The number of particle images in the respective orientation is depicted as color and the corresponding color key is given on the right.

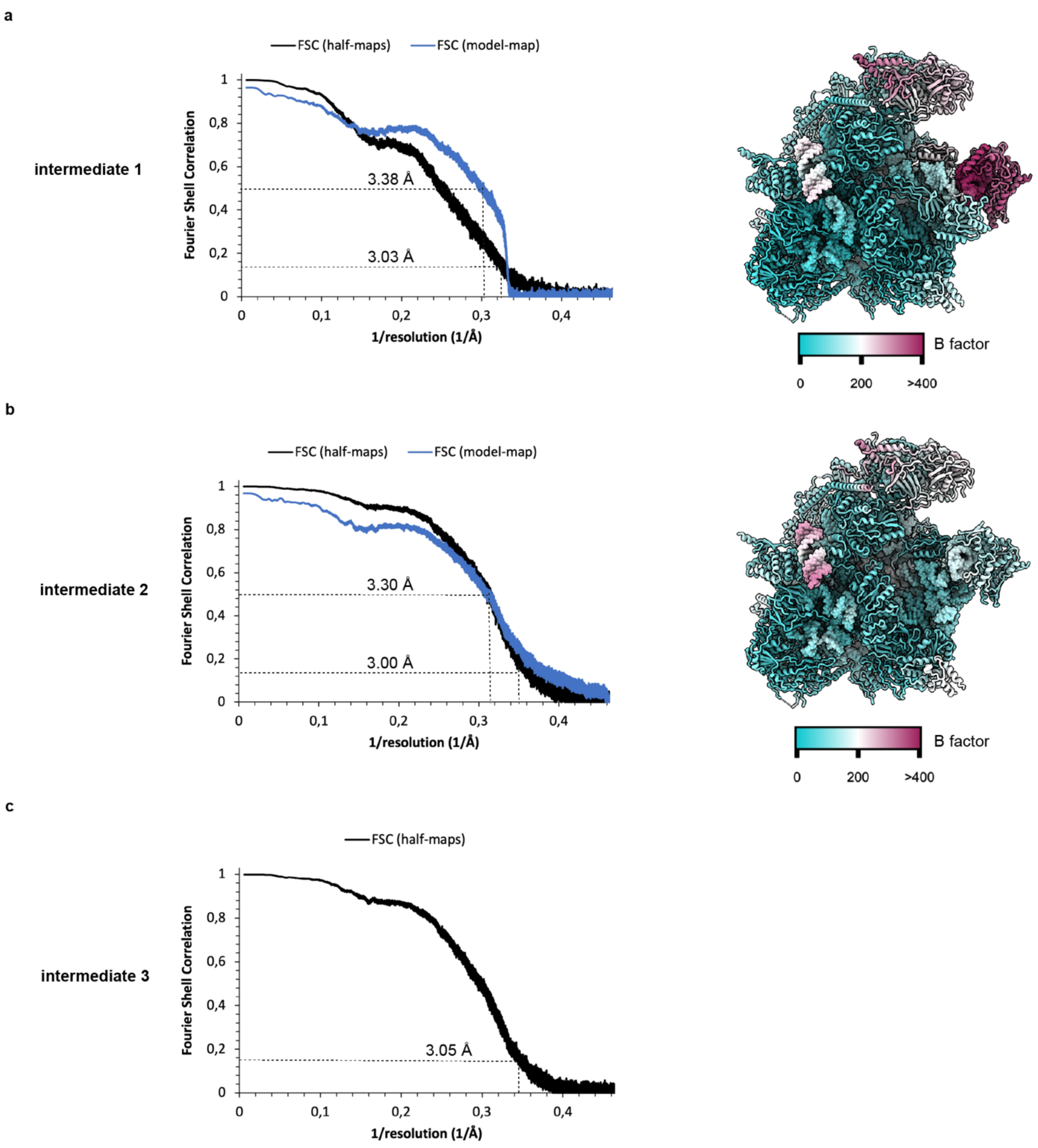

Supplementary Fig. 15 FSCs and B-factors

Data for intermediate 1, 2 and 3 are shown in panels **a)**, **b)**, and **c)**, respectively. The Fourier shell correlation (FSC) curves for half maps and model-to-map correlations have have been calculated in PHENIX using *phenix.mtriage*. For the half maps, the resolution cutoff at an FSC value of 0.143 is shown via the dashed line and the masked, overall resolution value is given in Å. In case of intermediate 1 and intermediate 2, the cutoff for the model-to-map FSC is shown at at 0.5 with a dashed line and the corresponding resolution value is given. The model is depicted as cartoon and coloured according to the B-factors as indicated by the colour key.

Supplementary Table 1: EM data collection and refinement statistics

**EM Data collection**			
Microscope model	FEI Titan Krios	FEI Titan Krios	FEI Titan Krios
Detector model	FEI Falcon 3C	FEI Falcon 3C	FEI Falcon 3C
Number of datasets	2	2	2
Number of micrographs collected	65’686	65’686	65’686
Pixel size (Å)	1.08 Å	1.08 Å	1.08 Å
Defocus range (µm)	0.4 - 2.8	0.4 - 2.8	0.4 - 2.8
Voltage (kV)	300	300	300
Electron dose ($e^{-}Å^{-2}$)	40	40	40
Name of 3D-reconstruction	intermediate 1	intermediate 2	intermediate 3
EMDB map entry	EMD-17719	EMD-17720	EMD-17721
PDB coordinate entry	PDB 8PK0	PDB 8QSJ	
Final number of particles	31’656	68’901	39’296
Resolution (Å) (at FSC = 0.143)	3.03	3.00	3.05
Map sharpening B-factor ($Å^2$)	-67.3	-78.1	-73.1
**Refinement and model validation statistics***			
Overall model geometry			
Clashscore (all atoms)	7.04	7.95	n/a
MolProbity score	1.41	1.45	n/a
Rmsd (bonds)	0.002	0.002	n/a
Rmsd (angles)	0.466	0.441	n/a
Ramachandran plot (%)			
favored	97.95	97.95	n/a
allowed	2.04	2.04	n/a
outliers	0.01	0.01	n/a
Rotamer outliers (%)	1.05	1.01	n/a
Cβ outliers (%)	0.00	0.00	n/a
Peptide plane (%)			
Cis proline/general	2.7/0.0	2.6/0.0	n/a
Twisted proline/general	0.2/0.0	0.2/0.0	n/a
CaBLAM outliers (%)	1.30	1.22	n/a
Resolution estimates (Å)			
FSC (half maps; 0.143)	3.03	3.0	n/a
FSC (model vs. full map; masked; 0.5)	3.38	3.3	n/a
CC (mask)	0.81	0.80	n/a
CC (box)	0.72	0.72	n/a

*The model was validated using MolProbity implemented in PHENIX Real-space refinement. n/a = not applicable

Supplementary References

1 Kummer, E., Schubert, K. N., Schoenhut, T., Scaiola, A. & Ban, N. Structural basis of translation termination, rescue, and recycling in mammalian mitochondria. *Mol Cell* **81**, 2566-2582 e2566 (2021). https://doi.org:10.1016/j.molcel.2021.03.042

2 Lenarcic, T. *et al.* Stepwise maturation of the peptidyl transferase region of human mitoribosomes. *Nat Commun* **12**, 3671 (2021). https://doi.org:10.1038/s41467-021-23811-8

3 Sekulski, K., Cruz, V. E., Weirich, C. S. & Erzberger, J. P. rRNA methylation by Spb1 regulates the GTPase activity of Nog2 during 60S ribosomal subunit assembly. *Nat Commun* **14**, 1207 (2023). https://doi.org:10.1038/s41467-023-36867-5

4 Nikolay, R. *et al.* Snapshots of native pre-50S ribosomes reveal a biogenesis factor network and evolutionary specialization. *Mol Cell* **81**, 1200-1215 e1209 (2021). https://doi.org:10.1016/j.molcel.2021.02.006

5 Cheng, J., Berninghausen, O. & Beckmann, R. A distinct assembly pathway of the human 39S late pre-mitoribosome. *Nat Commun* **12**, 4544 (2021). https://doi.org:10.1038/s41467-021-24818-x
