# Supplementary material for: Structural insights into the role of GTPBP10 in the RNA maturation of the mitoribosome
Source: Nat Commun. 2023 Dec 2;14:7991. doi: 10.1038/s41467-023-43599-z (PMC10693566; doi:10.1038/s41467-023-43599-z)
Supplement: Supplementary file 3 — Reporting Summary [file 41467_2023_43599_MOESM3_ESM.pdf]

nature portfolio

Corresponding author(s): Eva Kummer, NCOMMS-23-27420

Last updated by author(s): Nov 1, 2023

Reporting Summary

Nature Portfolio wishes to improve the reproducibility of the work that we publish. This form provides structure for consistency and transparency in reporting. For further information on Nature Portfolio policies, see our Editorial Policies and the Editorial Policy Checklist.

Statistics

For all statistical analyses, confirm that the following items are present in the figure legend, table legend, main text, or Methods section.

n/a	Confirmed	
☐	☒	The exact sample size (*n*) for each experimental group/condition, given as a discrete number and unit of measurement
☐	☒	A statement on whether measurements were taken from distinct samples or whether the same sample was measured repeatedly
☒	☐	The statistical test(s) used AND whether they are one- or two-sided<br>*Only common tests should be described solely by name; describe more complex techniques in the Methods section.*
☒	☐	A description of all covariates tested
☒	☐	A description of any assumptions or corrections, such as tests of normality and adjustment for multiple comparisons
☒	☐	A full description of the statistical parameters including central tendency (e.g. means) or other basic estimates (e.g. regression coefficient) AND variation (e.g. standard deviation) or associated estimates of uncertainty (e.g. confidence intervals)
☒	☐	For null hypothesis testing, the test statistic (e.g. *F*, *t*, *r*) with confidence intervals, effect sizes, degrees of freedom and *P* value noted<br>*Give P values as exact values whenever suitable.*
☒	☐	For Bayesian analysis, information on the choice of priors and Markov chain Monte Carlo settings
☒	☐	For hierarchical and complex designs, identification of the appropriate level for tests and full reporting of outcomes
☒	☐	Estimates of effect sizes (e.g. Cohen's *d*, Pearson's *r*), indicating how they were calculated

Our web collection on statistics for biologists contains articles on many of the points above.

Software and code

Policy information about availability of computer code

Data collection	EPU–Automated Acquisition Software (v2.14.0)
Data analysis	CryoSPARC v.4.2.0, Coot v.0.9.6, PHENIX v.1.19.2-4158, PyMOL v.2.5.5, UCSF Chimera v.1.15, ChimeraX v.1.2.5 and v.1.5

For manuscripts utilizing custom algorithms or software that are central to the research but not yet described in published literature, software must be made available to editors and reviewers. We strongly encourage code deposition in a community repository (e.g. GitHub). See the Nature Portfolio guidelines for submitting code & software for further information.

Data

Policy information about availability of data

All manuscripts must include a data availability statement. This statement should provide the following information, where applicable:

- Accession codes, unique identifiers, or web links for publicly available datasets
- A description of any restrictions on data availability
- For clinical datasets or third party data, please ensure that the statement adheres to our policy

Electron microscopy data have been deposited in the EMDB under accession codes EMD-17719 [https://www.ebi.ac.uk/emdb/EMD-17719] (intermediate 1), EMD-17720 [https://www.ebi.ac.uk/emdb/EMD-17720] (intermediate 2), and EMD-17721 [https://www.ebi.ac.uk/emdb/EMD-17721] (intermediate 3). The structural models for intermediate 1 and intermediate 2 have been deposited in the PDB database under the accession codes 8PK0 [http://doi.org/10.2210/

pdb8PK0/pdb] and 8QSJ [http://doi.org/10.2210/pdb8QSJ/pdb], respectively. Raw movies from cryo-EM analysis are available from the corresponding author upon request.

Research involving human participants, their data, or biological material

Policy information about studies with human participants or human data. See also policy information about sex, gender (identity/presentation), and sexual orientation and race, ethnicity and racism.

Reporting on sex and gender	N/A
Reporting on race, ethnicity, or other socially relevant groupings	N/A
Population characteristics	N/A
Recruitment	N/A
Ethics oversight	N/A

Note that full information on the approval of the study protocol must also be provided in the manuscript.

Field-specific reporting

Please select the one below that is the best fit for your research. If you are not sure, read the appropriate sections before making your selection.

☒ Life sciences ☐ Behavioural & social sciences ☐ Ecological, evolutionary & environmental sciences

For a reference copy of the document with all sections, see nature.com/documents/nr-reporting-summary-flat.pdf

Life sciences study design

All studies must disclose on these points even when the disclosure is negative.

Sample size	The sample size (number of micrographs and particles) was chosen such that it was high enough to allow for deep sorting of particle images and extraction of low-abundant endogenous ribosomal complexes from the ribosomal pool.
Data exclusions	Poor micrographs with high ice contamination, poor resolution, or poor particle distribution were excluded from the analysis. After 2D and 3D classification, all particle images were excluded that did not yield clear 2D classes or that corresponded to 3D classes of either already interpreted ribosomal complexes or LSU substates with no maturation factors bound.
Replication	Data were not replicated as the final reconstruction is yielded from many particles of the same maturation state that were computationally isolated from the bulk population.
Randomization	Data are randomized multipe times throughout the cryo-EM data processing workflow at the onset of 2D, 3D classification, and during 3D refinement.
Blinding	Data processing did not involve blinding as all particle images extracted from the micrographs entered the classification process.

Reporting for specific materials, systems and methods

We require information from authors about some types of materials, experimental systems and methods used in many studies. Here, indicate whether each material, system or method listed is relevant to your study. If you are not sure if a list item applies to your research, read the appropriate section before selecting a response.

Materials & experimental systems

n/a	Involved in the study	
☒	☐	Antibodies
☐	☒	Eukaryotic cell lines
☒	☐	Palaeontology and archaeology
☒	☐	Animals and other organisms
☒	☐	Clinical data
☒	☐	Dual use research of concern
☒	☐	Plants

Methods

n/a	Involved in the study	
☒	☐	ChIP-seq
☒	☐	Flow cytometry
☒	☐	MRI-based neuroimaging

Eukaryotic cell lines

Policy information about cell lines and Sex and Gender in Research

Cell line source(s)	HEK293-EBNA1-6E cells were obtained from the Protein Production and Characterization Platform at the Novo Nordisk Foundation Center for Protein research, University of Copenhagen.
Authentication	The cell line was not authenticated.
Mycoplasma contamination	Cells were not tested for Mycoplasma contamination.
Commonly misidentified lines (See ICLAC register)	No misidentified cell lines were used.
